# Factors Predicting Response to the Recovery-Oriented Cognitive Behavioural Workshop for Persons Diagnosed with Schizophrenia

**DOI:** 10.1007/s10597-020-00595-7

**Published:** 2020-04-01

**Authors:** Izabela Nowak, Piotr Świtaj, Cornelia Oberhauser, Marta Anczewska

**Affiliations:** 1 First Department of Psychiatry, Institute of Psychiatry and Neurology, Sobieskiego 9, 02-957 Warsaw, Poland; 2 Institute for Medical Information Processing, Biometry, and Epidemiology – IBE, Chair of Public Health and Health Services Research, LMU Munich, Marchioninistr. 17, 81377 Munich, Germany

**Keywords:** Schizophrenia, Disability, Personal recovery, Cognitive behavioural therapy

## Abstract

A recovery-oriented, cognitive behavioural workshop for service users diagnosed with schizophrenia was developed, implemented and evaluated in a pilot study. Further analysis is required regarding factors which contribute to better treatment response, as this will provide useful information for workshop adaptation. Secondary multilevel model analyses were performed to determine whether workshop and booster session attendance, as well as sociodemographic variables such as gender, age, education, and duration of illness, predicted workshop responsiveness. Results showed that completers had lower responsiveness to the workshop in terms of confidence and hope, whereas those who attended an online booster session demonstrated better responsiveness as to psychosocial functioning. Longer duration of illness and older age generally predicted lower intervention responsiveness. In conclusion, adaptations utilising more booster sessions and accommodating older participants with longer duration of illness are required, as is further workshop evaluation in a randomised controlled study.

## Introduction

Individuals diagnosed with schizophrenia experience disability in their daily lives. Using the criteria of the International Classification of Functioning Disability and Health (ICF) (WHO 2001) these refer not only to impairments of mental functions but also to several activities and participation domains. The most extensively reported in the literature amongst mental functions are cognitive and emotional functions. Within the domain of activities and participation, difficulties in relationships with others and employment (Świtaj et al. 2012). Environmental factors (e.g. frustrations with mental health services, stigmatising societal attitudes and personal factors) further impact the level of disability experienced (Hartley et al. 2014). Therefore, there is a need to develop interventions that support the real-world functioning of persons diagnosed with schizophrenia (Nowak et al. 2016).

The recovery approach recognises that the needs of persons experiencing mental health problems go beyond symptomatic reduction by encouraging their self-determination and engagement in life pursuits (Anthony 1993; Farkas 2007). Some key components of recovery identified by service users in recovery include regaining hope, empowerment, social connection, having meaning and purpose in life, transformation of identity, re-assuming responsibility and control, managing symptoms and combating stigma (Schrank and Slade 2007).

Relevant processes of personal recovery synthesised in a systematic literature review and named as the CHIME framework included connectedness, hope and optimism about the future, identity, meaning in life, and empowerment (Leamy et al. 2011). In terms of treatment, empirically supported pro-recovery interventions have been reported (Slade et al. 2014). However, although CBT for psychosis (CBTp) ranks amongst the psychosocial interventions recommended for routine practice (National Institute for Health and Care Excellence 2014), only a few recovery-focused cognitive behavioural interventions are available for schizophrenia (Nowak et al. 2016).

In response to those needs, a recovery-oriented, cognitive behavioural workshop for persons diagnosed with schizophrenia was developed to support the personal recovery and real-world functioning of service users. The workshop fits into literature on empirically-supported recovery-oriented interventions (e.g. Grant et al. 2012) in terms of integrating the principles and spirit of the recovery movement in the intervention conceptualization. However, it also takes into account cultural aspects relevant to personal recovery and is grounded in the life-long learning approach, meaning that it is not a psychotherapeutic intervention but an educational workshop.

Results of the first evaluation revealed the workshop is a promising intervention with potential in terms of both improving personal recovery and real-life functioning of persons diagnosed with schizophrenia. Nevertheless, at this stage of intervention development, it is crucial to evaluate which factors contributed to better treatment response. Lack of clarity in this respect may result in a possible mismatch between the delivered intervention and the identified population’s specific needs. Results of a systematic review by O'Keeffe et al. (2017) have shown that CBTp does not work equally well for everyone: for instance, female gender, older age (> 21), a shorter duration of the illness, and higher educational attainment predicted better outcomes in CBT interventions with psychotic patients. Furthermore, the development of new interventions involves using the best available evidence and theory as well as testing interventions in phased approaches that begin with pilot studies and progress to definitive evaluation (Craig et al. 2008).

Therefore, this study aims to explore factors of treatment response, which will facilitate the adaptation of the workshop manual before evaluating it in a randomised controlled trial. Particularly, it was foreseen to evaluate whether the workshop and booster session attendance, as well as sociodemographic characteristics (gender, age, level of education, and duration of illness), predicted workshop responsiveness.

## Methods

### Study Overview and Design

The intervention was evaluated in an uncontrolled, pre-post design. Participants were assessed at baseline prior to the commencement of the workshop (T = 0), at the end of the workshop (T = 1), and at 1-month follow-up (T = 2). Recruitment took place in the facilities of the Institute of Neurology and Psychiatry in Warsaw (an outpatient clinic and a relapse prevention ward). The study was approved by the Bioethical Committee at the Institute of Psychiatry and Neurology. All participants signed an informed consent form prior to the commencement of the study.

### Participants

The inclusion criteria of participants consisted of the following: schizophrenia diagnosis (F20, the International Classification of Diseases, ICD-10), 18–65 years of age and stable mental health status according to the treating psychiatrist, enabling participation in the workshop. The exclusion criteria were acute psychosis, withdrawal of consent to participate in the study, no diagnosis of schizophrenia and active drug or alcohol dependence. Forty-six participants consented to take part in the study (*N* = 46). Participants ranged in age from 19 to 61 years (*M* = 32.89, *SD* = 9.00). There were 16 participants (*M* = 24.38, *SD* = 3.16) in the 19–29 age group and 30 (*M* = 37.43, *SD* = 7.69) in the 30–61 age group. The cut-off age of 29 for the younger reference group was adapted from Edwards and McGorry (2002). Most participants were men (*n* = 24, 52.2%). Twenty-five (54.3%) participants had a higher education and 21 (45.7%) individuals had primary or secondary school education. The mean duration of illness was 7.54 years (*SD* = 7.64) for the total sample, 1.75 (*SD* = 1.94) years for 21 participants (45.7%) who were in the first 5 years of illness and 12.4 (*SD* = 7.23) years for 25 (54.3%) individuals whose mean duration of illness was more than 5 years. Twenty-nine participants (63%) attended all seven workshop modules, and 12 (21.1%) attended the booster session. Non-completers vs. completers had a higher mean score on total recovery (RAS-R) (*M* = 89.65, *SD* = 13.20 vs *M* = 79.79 *SD* = 14.16) and on personal confidence and hope subscale (*M* = 33.18 *SD* = 5.01 vs *M* = 28.55 *SD* = 6.49) at baseline. These differences were statistically significant with t(44) = 2.335, p < .024 for total recovery score and t(44) = 2.525, p < .015 for personal confidence and hope.

### Measures

Outcome measures evaluated personal recovery, psychosocial difficulties, severity of psychopathological symptoms and health-related behaviours. Recovery was measured with both the total score and factors of the short version of the Recovery Assessment Scale-Revised (RAS-R; Corrigan et al. 2004). It uses a five-point Likert scale to measure the extent of agreement, ranging from 1 = *strongly disagree* to 5 = *strongly agree,* with higher scores indicating improved recovery. The RAS-R factors are personal confidence and hope, willingness to ask for help, goal and success orientation, reliance on others and no domination by symptoms, with Cronbach’s alpha for the subscales ranging from 0.74 to 0.87.

Psychosocial functioning was evaluated with the PARADISE 24 metric. It assesses the impact of mental and neurological health conditions on people’s lives and estimates individuals’ overall level of disability, termed *psychosocial difficulties* (PSDs). It contains 24 items scored on a three-point scale (0 = *none*, 1 = *some*, 2 = *a lot*). Total scores, acquired from the sum of PSD scores, range from 0 (*no PSDs*) to 100 (*extreme PSDs*). Rash analyses indicated a valid, reliable metric, with questions infit mean squares ranging from 0.7 to 1.3. Targeting between item thresholds and persons’ abilities was good, whereas the person separation index was 0.92 (Cieza et al. 2015).

The severity of psychopathological symptoms was assessed with the Brief Psychiatric Rating Scale (BPRS; Overall and Gorham 1988). It comprises 18 clinician-rated items with scoring options ranging from 1 (*symptom not present*) to 7 (*symptom extremely severe*). Adding up individual responses gives a total scale score, which can range from 18 to 126, with higher scores indicating more severe symptoms.

Health-related behaviours were measured by the Inventory of Health Behaviours (Inwentarz Zachowań Zdrowotnych; Juczyński 2012). It evaluates various health-related behaviours, including proper nutritional habits, preventive behaviours, positive thinking and health practices. Responses range from 1 = *almost never* to 5 = *almost always*. Higher scores indicate a greater intensity of healthy behaviours. Cronbach’s alpha for the whole questionnaire is 0.85, whereas the rates for its subscales range from 0.60 to 0.65. All questionnaires are self-reports except for BPRS, which was filled out by treating psychiatrists.

### Recovery-Oriented Cognitive Behavioural Workshop

The workshop was developed based on results from the systematic literature review (Nowak et al. 2016) indicating that there is a need to develop more recovery-oriented interventions and a focus group study (Nowak et al. 2017) that guided the development of workshop modules. As psychological recovery was the most widely supported theme, the first three modules focused on personal definitions of recovery, strengthening positive sense of self, and value-based goal setting, whereas the content of further modules referred to more objective recovery domains, such as self-management of mental and physical health as well as enhancing relationships with others. Traditional and ‘third wave’ CBT techniques were used to foster participants’ knowledge about personal recovery. Feedback on the workshop was sought from experts in the recovery approach and in CBT therapy.

The first workshop module aimed to facilitate reflection about the concept of personal recovery as well as the personal recovery journey. Module two revolved around identity change in the context of illness appearance as well as fostering personal strengths and capacities. Module three facilitated identification of personal values and life goals related to them. Modules four and five were aimed at increasing participants’ sense of self-efficacy in dealing with mental health problems as well as developing strategies to support healthy behaviours, such as following a balanced diet, physical activity and looking after one’s health. Modules six and seven focused on developing the skills necessary to initiate and develop friendships, disclosure of mental health illness and assertive ways of asking for help. Two weeks after the treatment termination, an optional online booster session was offered to participants. It aimed to follow up the participants’ progress toward recovery as well as boost motivation and confidence in overcoming difficulties. A recovery workbook was distributed to participants; sessions were guided by PowerPoint presentations. More detailed description of the workshop was provided in Table S1 in the supplementary material of the study published by Nowak et al. (2019).

### Overview of Data Analysis

Multilevel models of change were run for each dependent variable and incorporated a level-1 submodel describing the general trend of change over time and a level-2 model describing how these changes differ across people. Results of the level-1 submodel for each dependent variable were reported by Nowak et al. (2019) and revealed that the total recovery score (RAS-R) was not significant; however, participants improved over time regarding confidence and hope and feeling less dominated by symptoms (RAS-R subscales). Likewise, participants improved in psychosocial functioning and psychopathology. In this study, we aimed to fit a level-2 submodel to the variables that were statistically significant in the first evaluation by introducing further predictors to the analysis. The following predictors were fitted in the level-2 model: workshop and booster session attendance, gender, age, education and duration of illness. Age and duration of illness were tested as both metric and categorical variables. Model fit was examined based on the Akaike information criterion (AIC). Sociodemographic variables that resulted in a worse model fit based on AIC were excluded from further analysis. Analyses were performed on participants who answered the questionnaires at two time points as a minimum, with the SPSS MIXED procedure of SPSS version 23.

## Results

Considering the dependent variable personal confidence and hope (RAS-R), model fitting including workshop attendance [F (1,48.67) = 5.148, *p* < .028], booster session [F (1,39.68) = 3.145, *p* < .084] and age [F (1,41.83) = 3.168, *p* < .082] as predictors indicated that the effect of the group booster session attendees compared to the group non-full-attendees had a trend towards higher workshop responsiveness, but the effect of the group older participants compared to the group younger participants had a trend toward lower workshop responsiveness.

Final model fitting including wokshop attendance [F (1,49.856) = 4.453, *p* < .040], booster session [F (1,39.69) = 2.581, *p* > .116], age [F (1,42.126) = 0.624, *p* > .434], and duration of illness [F (1,45.38) = 3.282, *p* < .077] revealed that significantly less responsiveness to the workshop was observed amongst the group workshop completers when compared to the group non-full-attendees. In addition, the effect of increased longer duration of illness (in years) predicted a negative trend toward less workshop responsiveness.

In terms of the no domination by symptoms (RAS-R) variable, final model fitting including workshop attendance [F (1,46.069 = 0.991, *p* > .325], booster session [F (1,37.930 = 0.120, *p* > .731] and duration of illness [F (1,42.76 = 3.849, *p* < .056] revealed that the effect of increased longer duration of illness (in years) predicted a borderline significant less responsiveness to the workshop. Neither workshop completers nor attending the booster session significantly predicted responsiveness to the workshop.

The analysis of psychosocial difficulties (PARADISE 24) with final model fitting including workshop attendance[F (1,47.736) = 1.897, *p* > .175], booster session [F (1,29.962) = 4.453, *p* < .043], age [F (1,39.547) = 10.845, *p* < .002] and duration of illness [F (1,37.739) = 5.276, *p* < .027] showed that participation in the booster session predicted a decrease in psychosocial difficulties, whereas the effect of increased older age (in years) and the effect of the group comparison (longer duration versus shorter duration of illness) predicted an increase in psychosocial difficulties. Finally, none of the variables predicted *symptomatology* (BPRS); therefore, no model fitting proceeded for this outcome. Gender and education did not predict any of the dependent variables, therefore, they were removed from the analysis.

## Discussion

This study aimed to evaluate factors predicting response to the recovery-oriented, cognitive behavioural workshop for persons diagnosed with schizophrenia. Specifically, it assessed whether the workshop and booster session attendance, as well as sociodemographic characteristics (gender, age, level of education, and duration of illness), predicted workshop responsiveness.

Firstly, the results revealed that, regarding the variable personal confidence and hope, completers had less responsiveness to the workshop when compared to participants who did not attend all sessions. A similar trend, although not statistically significant, was observed for the other dependent variables. This is a surprising result. However, considering that there was a statistically significant improvement regarding the variable personal confidence and hope over time, these results might mean that participants who took part in all workshop modules did not improve as much as participants who did not attend all sessions. It also indicates that non-completers had higher scores when compared to completers in respect to personal recovery, therefore their earlier workshop termination.

There was an overall trend for better workshop responsiveness among booster session attendees. Obtained results are consistent with studies which indicate that providing booster sessions or maintaining treatment after the conclusion of CBT treatment are beneficial in terms of long-term effects (Stangier et al. 2013). These findings have direct implications for the workshop’s manual improvement, as booster sessions might provide a useful way to deliver the workshop over a more extended period. This is particularly important since further work is needed in various areas related to recovery as reported in the study by Nowak et al. (2019).

O’Keeffe et al. (2017), exploring factors that predict favourable outcome in cognitive behavioural interventions for psychosis, indicated that female gender, higher educational status, and shorter duration of illness were predictive of better outcome in CBT interventions. The results of this study show that the workshop was equally responsive regardless of participants’ gender or educational status. However, older participants with longer duration of illness had poorer responsiveness to the workshop. This indicates the need for adapting the workshop for middle-aged and older service users diagnosed with schizophrenia.

Obtained results are not surprising, older service users with psychosis might have experienced difficulties, which can lead to low expectations of success. Information-processing biases may additionally lead to a focus on failures while ignoring past achievements. Also, by this stage of illness, older individuals with psychosis have possibly been exposed to stigma related to low chances of recovery. These factors may present unique challenges met less frequently when working with younger service users. However, there is a good clinical reason to believe that CBT interventions can be successful in reducing distress and disability among older people with psychosis if adapted appropriately (Kingdon et al. 2008).

In conclusion, booster sessions might provide a useful way for further workshop delivery since more work is needed in some areas related to recovery. The workshop seems more suited for younger participants with shorter duration of illness. Therefore, if delivered to middle-aged or older participants, some adaptations are required. A study into the concept of recovery among older Polish people with schizophrenia might shed light on recovery components relevant to this age group. Daley et al. (2013) reported that the impact of illness, the significance of personal responsibility, and specific coping strategies were of importance to older people with mental disorders. These aspects differed from younger peers, who sought to re-establish a new sense of identity as well as support from others with lived experience of mental illness.

The main limitation of the study was its uncontrolled, pre-post design and short follow-up. Furthermore, the size of our sample may not have been large enough to detect small effects. Small sample size also limited the number of predictors included in the analysis. Finally, we have only assessed global severity of psychiatric symptoms, without considering specific symptom dimensions. The uncontrolled study design significantly limits the conclusions and precludes any causal inferences about the workshop efficacy. Future research could incorporate randomisation to an appropriate control condition with a longer follow-up, which would improve the reliability and generalisability of findings.
